# Ling Zhi-8, a fungal immunomodulatory protein in *Ganoderma lucidum*, alleviates CPT-11-induced intestinal injury via restoring claudin-1 expression

**DOI:** 10.18632/aging.204695

**Published:** 2023-05-05

**Authors:** Ju-Pi Li, Ching-Liang Chu, Wan-Ru Chao, Cheng-Siang Yeh, Yi-Ju Lee, Dz-Chi Chen, Shun-Fa Yang, Yu-Hua Chao

**Affiliations:** 1 Department of Pediatrics, Chung Shan Medical University Hospital, Taichung, Taiwan; 2 School of Medicine, Chung Shan Medical University, Taichung, Taiwan; 3 Graduate Institute of Immunology, College of Medicine, National Taiwan University, Taipei, Taiwan; 4 Department of Pathology, Chung Shan Medical University Hospital, Taichung, Taiwan; 5 Yeastern Biotech Co., Ltd., New Taipei City, Taiwan; 6 Department of Medical Research, Chung Shan Medical University Hospital, Taichung, Taiwan; 7 Institute of Medicine, Chung Shan Medical University, Taichung, Taiwan; 8 Department of Clinical Pathology, Chung Shan Medical University Hospital, Taichung, Taiwan

**Keywords:** claudin-1, CPT-11, Ganoderma lucidum, irinotecan, Ling Zhi-8

## Abstract

CPT-11 (Irinotecan) remains an important chemotherapeutic agent against various solid tumors nowadays. Potential adverse effects, especially gastrointestinal toxicities, are the main limiting factor for its clinical utility. Ling Zhi-8 (LZ-8), a fungal immunomodulatory protein in *Ganoderma lucidum* mycelia, has potential for drug development due to its multiple bioactivities and functions. This study aimed to explore the influence of LZ-8 on CPT-11-treated IEC-6 cells *in vitro* and on mice with CPT-11-induced intestinal injury *in vivo*. The mechanism through which LZ-8 exerted its protective effects was also investigated. In the *in vitro* study, the viability and claudin-1 expression of IEC-6 cells decreased gradually with increasing concentrations of CPT-11, but LZ-8 treatment had no obvious influence on their viability, morphology, and claudin-1 expression. Pretreatment of LZ-8 significantly improved CPT-11-decreased cell viability and claudin-1 expression in IEC-6 cells. In mice with CPT-11-induced intestinal injury, LZ-8 treatment could ameliorate symptoms and mitigate intestinal damage. Meanwhile, LZ-8 restored claudin-1 expression in the intestinal membranes in CPT-11-treated mice. Collectively, our results demonstrated the protective effects of LZ-8 against CPT-11 damage in both IEC-6 cells and mice. LZ-8 can restore claudin-1 expression in intestinal cells following CPT-11 treatment, suggesting the role of claudin-1 in the scenario.

## INTRODUCTION

Also known as irinotecan, CPT-11 has largely contributed to treat metastatic or advanced solid tumors, such as colon, pancreatic, ovarian and lung cancers. CPT-11 is a plant alkaloid isolated from the Chinese tree *Camptotheca acuminata*, and was first approved for the treatment of cancer in 1994 [1]. Being an anticancer agent, CPT-11 belongs to the class of topoisomerase I inhibitors, acting on S and G2 phases of the cell cycle. Treatment with CPT-11 is often accompanied by various toxicities, primarily neutropenia and gastrointestinal discomforts [2]. CPT-11-associated diarrhea is the most common dose-limiting toxicity and can be life-threatening. Several approaches, such as a combination use of loperamide or octreotide, have been attempted to minimize its adverse effects and to improve the tolerance of increasing doses. However, loperamide is associated with a significant failure rate and beneficial effects of octreotide are controversial in clinical practice [3]. Therefore, the medical community should put in efforts continuously to make CPT-11 more tolerable during treatment.

Ling Zhi (*Ganoderma lucidum*), the most famous medicinal mushroom, has been used in traditional medicine of the Far East for more than 2000 years. Being a traditional herb with miracle cure and health promoting benefits, modern scientific evaluations also have demonstrated the anti-inflammatory, anti-oxidant, anti-cancer, anti-bacterial, anti-viral, immunomodulatory, hepato-protective, nephron-protective, healing promotion, and anti-aging effects of *Ganoderma lucidum* and its extracts [4, 5]. Ling Zhi-8 (LZ-8), which is a component in *Ganoderma lucidum* mycelia [6], belongs to the family of fungal immunomodulatory proteins. The complete amino acid sequence and crystal structure of LZ-8 has been illustrated [7, 8]. Along with a growing body of related investigations, it is useful for enhancing its utility both in the fields of health food and alternative medicine.

Despite having a series of documented bioactivities, it remains unknown if LZ-8 can alleviate CPT-11-induced intestinal toxicity. In the present study, we aimed to determine the effects of LZ-8 on CPT-11-treated IEC-6 cells *in vitro* and on mice with CPT-11-induced intestinal injury *in vivo*. The mechanism through which LZ-8 exerted its protective effects was also investigated.

## RESULTS

### Protective effects of LZ-8 against CPT-11-induced cytotoxicity in IEC-6 cells

**Figure 1:**
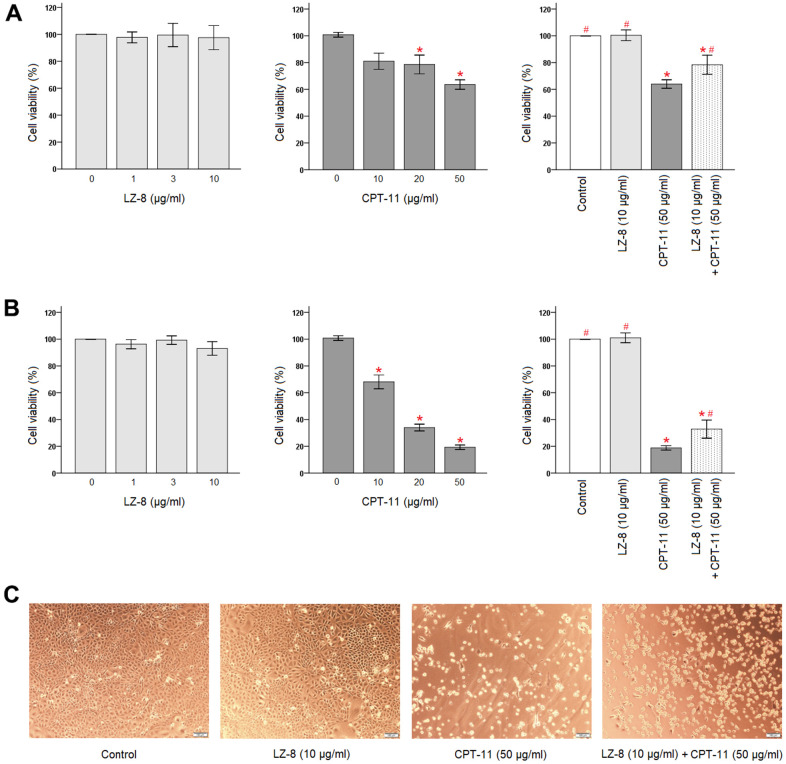
**Protective effects of LZ-8 against CPT-11-induced cytotoxicity in IEC-6 cells.** CCK-8 assay was used to determine the viability of IEC-6 cells. For control, the absorbance of untreated cells was considered 100%. LZ-8 treatment had no obvious influence on the viability and morphology of IEC-6 cells, but cell death in a dose-dependent manner was noted in IEC-6 cell treated with CPT-11. LZ-8 pretreatment improved the cell viability and morphology of IEC-6 cells following CPT-11 treatment. Data were presented as mean ± SD, and one-way ANOVA was used for statistical analysis. * indicates *P* < 0.05, compared with the untreated cells; # indicates *P* < 0.05, compared with the CPT-11-treated cells. n = 5. (**A**) The viability of IEC-6 cells after 24-hour incubation of LZ-8 (left panel), 24-hour incubation of CPT-11 (middle panel), or 24-hour pretreatment of LZ-8 following 24-hour incubation of CPT-11 (right panel). (**B**) The viability of IEC-6 cells after 48-hour incubation of LZ-8 (left panel), 48-hour incubation of CPT-11 (middle panel), or 24-hour pretreatment of LZ-8 following 48-hour incubation of CPT-11 (right panel). (**C**) Effects of LZ-8 on CPT-11-induced cell morphological changes.

First, effects of LZ-8 and CPT-11 on the viability of IEC-6 cells were determined by Cell Counting Kit-8 (CCK-8) (Figure 1). LZ-8 treatment at concentrations of 1, 3, and 10 μg/ml had no obvious influence on the viability and morphology of IEC-6 cells, compared to the untreated cells. The time of LZ-8 treatment, 24 or 48 hours, appeared to have similar results. In contrast, cell death in a dose-dependent manner was noted in IEC-6 cells treated with CPT-11 for 24 hours (Figure 1A, middle panel). Compared with the untreated cells, the viability of IEC-6 cells decreased gradually with increasing concentrations of CPT-11 (*P* < 0.05). The cell viability was 63.6 ± 3.5% following incubation with 50 μg/ml CPT-11 for 24 hours. The impact of CPT-11 was augmented markedly by increasing the incubation time to 48 hours (Figure 1B, middle panel). The dose-dependent cytotoxicity of CPT-11 on IEC-6 cells was more evident, and the cell viability declined to 19.3 ± 1.7% following incubation with 50 μg/ml CPT-11 for 48 hours. In addition to the decrease in the viable cell number, significant morphological changes were also observed in CPT-11-treated IEC-6 cells (Figure 1C). The cells were shrunken and altered from fusiform to polygonal shape.

Second, protective effects of LZ-8 pretreatment on CPT-11-induced cytotoxicity in IEC-6 cells were examined. As shown in Figure 1A (right panel), 24-hour pretreatment of 10 μg/ml LZ-8 significantly improved the cell viability of IEC-6 cells following incubation with 50 μg/ml CPT-11 for 24 hours, compared to the cells treated with CPT-11 alone (78.4 ± 7.1% vs 64.0 ± 3.2%; *P* = 0.028). The beneficial effects of LZ-8 pretreatment on CPT-11-induced cell death remained evident when incubation with CPT-11 for 48 hours (Figure 1B, right panel; 32.8 ± 6.8% vs 18.8 ± 4.6%; *P* = 0.029). As observed in Figure 1C, LZ-8 pretreatment maintained the normal morphology of IEC-6 cells to a certain extent.

### Effects of LZ-8 pretreatment on claudin-1 expression in CPT-11-treated IEC-6 cells

**Figure 2:**
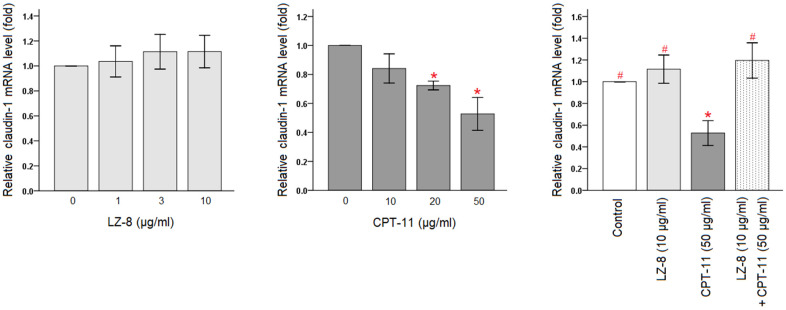
**Restoration of claudin-1 mRNA expression by pretreatment of LZ-8 in IEC-6 cells following CPT-11 treatment, assessed by qPCR analysis.** After normalization with β-actin, relative expression levels were shown with the mean value of untreated cells in the control group set at 1. LZ-8 treatment had no obvious influence on the expression levels of claudin-1 mRNA in IEC-6 cells, but CPT-11-treated IEC-6 cells expressed lower levels in a dose-dependent manner. LZ-8 pretreatment increased claudin-1 mRNA expression in IEC-6 cells following CPT-11 treatment. Data were presented as mean ± SD, and one-way ANOVA was used for statistical analysis. * indicates *P* < 0.05, compared with the untreated cells; # indicates *P* < 0.05, compared with the CPT-11-treated cells. n = 5.

The protective effects of LZ-8 on CPT-11-induced cytotoxicity in IEC-6 cells were confirmed, and the molecular mechanism involving the function of LZ-8 was further investigated. As claudin-1 is an important tight junction protein for the maintenance of intestinal barrier function, we determined the expression levels of claudin-1 in IEC-6 cells following different treatments by quantitative polymerase chain reaction (qPCR). As shown in Figure 2, LZ-8 treatment at any concentrations did not markedly alter the expression levels of claudin-1 mRNA in IEC-6 cells, compared to the untreated cells. However, CPT-11-treated IEC-6 cells exhibited lower expression of claudin-1 mRNA in a dose-dependent manner. Compared with the untreated cells, its expression decreased gradually with increasing concentrations of CPT-11 (*P* < 0.05). The relative levels declined to 0.53 ± 0.11 following incubation with 50 μg/ml CPT-11 for 24 hours (*P* = 0.003). It is worth noting that 24-hour pretreatment of 10 μg/ml LZ-8 significantly increased claudin-1 mRNA expression in IEC-6 cells following 24-hour incubation with 50 μg/ml CPT-11, compared to the cells treated with CPT-11 alone (1.20 ± 0.16 vs 0.53 ± 0.11; *P* = 0.001).

**Figure 3:**
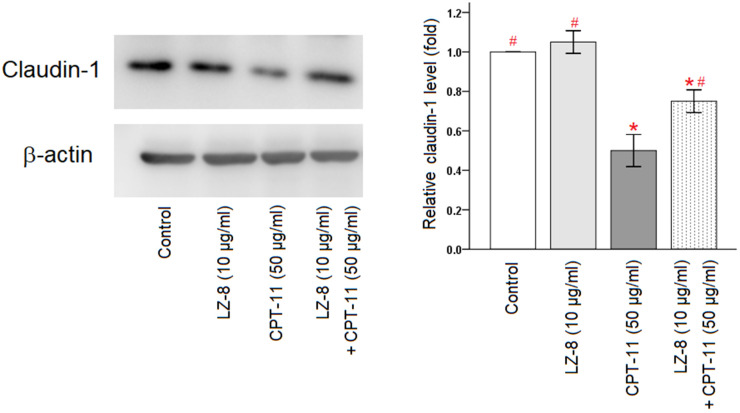
**Improvement of claudin-1 protein expression by pretreatment of LZ-8 in IEC-6 cells following CPT-11 treatment, assessed by Western blotting.** After normalization with β-actin, relative expression levels were shown with the mean value of untreated cells in the control group set at 1. LZ-8 treatment had no obvious influence on the expression levels of claudin-1 protein in IEC-6 cells, but CPT-11-treated IEC-6 cells expressed lower levels. LZ-8 pretreatment increased claudin-1 protein expression in IEC-6 cells following CPT-11 treatment. Data were presented as mean ± SD, and one-way ANOVA was used for statistical analysis. * indicates *P* < 0.05, compared with the untreated cells; # indicates *P* < 0.05, compared with the CPT-11-treated cells. n = 4.

Furthermore, claudin-1 protein expression in IEC-6 cells following different treatments were analyzed by Western blotting (Figure 3 and Supplementary Figure 1). Compared with the untreated cells, incubation with LZ-8 did not have obvious influence on the expression levels of claudin-1 protein in IEC-6 cells but incubation with CPT-11 markedly decreased the expression levels. Consistent with results of the qPCR analysis, pretreatment of LZ-8 significantly improved the expression of claudin-1 protein in IEC-6 cells following CPT-11 treatment, compared to the cells treated with CPT-11 alone (0.75 ± 0.06 vs 0.50 ± 0.08; *P* = 0.013). Our results suggested that LZ-8 may exert the protective effects on CPT-11-induced injury in IEC-6 cells via restoring claudin-1 expression.

### Protective effects of LZ-8 on CPT-11-induced diarrhea and body weight loss in mice

During the first four days, mice of the LZ and LZ+CPT groups were fed with LZ-8 and mice of the control and CPT groups received oral phosphate-buffered saline (PBS) as controls. Mice in all four groups shared similar general conditions, including food intake and activity. Normal feces conditions were observed in all mice, and no obvious difference in body weight gain was noted between the groups. These results suggested that short-term use of LZ-8 did not have significant impact on the mice.

Four consecutive daily doses of 75 mg/kg CPT-11, starting from day 5 to day 8, was used to induce intestinal injury in mice. Compared to mice receiving PBS injections in the control and LZ groups, symptoms of fatigue, malaise, and anorexia became apparent day by day, more markedly in mice of the CPT group than those of the LZ+CPT group. Diarrhea was observed after two injections of CPT-11 in half of the mice, and all mice had diarrhea after three injections. As shown in Table 1, the severity of diarrhea was attenuate in mice treated with LZ-8 compared to those receiving oral PBS only in the CPT group, with significantly lower scores on day 8 and day 9 (*P* < 0.05). Watery diarrhea (grade 3) was noted on day 9 in three mice of the CPT group, but none of the LZ+CPT group. Fecal occult blood was not detected until day 9, with positive in all mice of the CPT group and only two in the LZ+CPT group (*P* = 0.011). Feces conditions in mice of the control and LZ groups remained normal and unchanged. Our results implicated that LZ-8 treatment could ameliorate symptoms of CPT-11-induced intestinal injury in mice.

**Table 1:** Severity of diarrhea and grading of fecal occult blood in mice.

**Group**	**Average diarrhea score**				**Diarrhea score on day 9 (n = 5)**					**Occult blood score on day 9 (n = 5)**		
	**Day 7**	**Day 8**	**Day 9**		**Grade 0**	**Grade 1**	**Grade 2**	**Grade 3**		**0**	**Trace**	**1+**
Control	0	0^#^	0^#^		5	0	0	0		5	0	0
LZ	0	0^#^	0^#^		5	0	0	0		5	0	0
CPT	0.6 ± 0.5	1.6 ± 0.5^*^	2.6 ± 0.5^*^		0	0	2	3		0	1	4
LZ+CPT	0.4 ± 0.5	1.0 ± 0.0^*#^	1.6 ± 0.5^*#^		0	2	3	0		3	2	0

Average diarrhea score data were presented as mean ± SD; chi-square test was used for statistical analysis. ^*^P < 0.05, compared with the control group. ^#^*P* < 0.05, compared with the CPT group.

**Figure 4:**
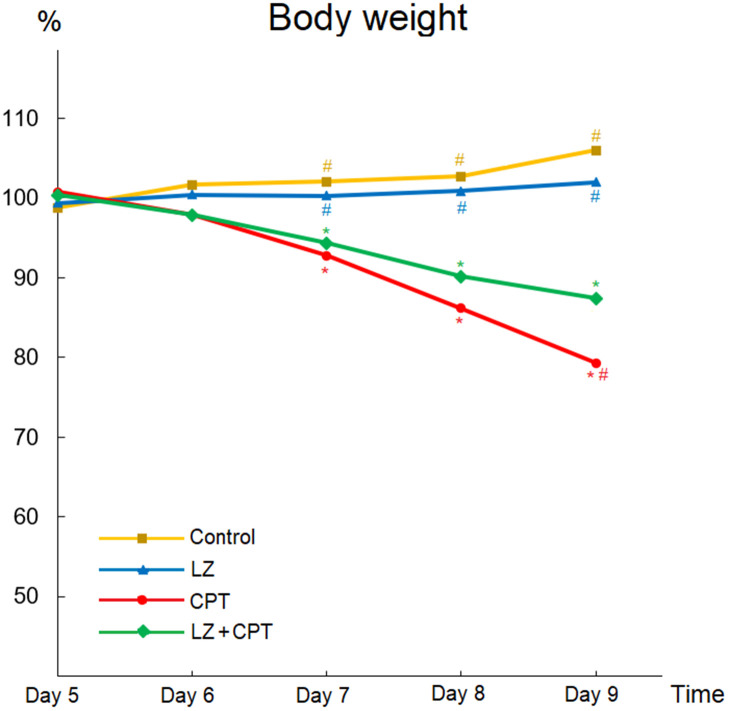
**Beneficial effects of LZ-8 on body weight loss in mice with CPT-11-induced intestinal injury.** Relative weight was calculated for each animal relative to its weight on day 4. There were no apparent changes of body weight profiles in the control and LZ groups, but body weights decreased significantly in mice of the CPT and LZ+CPT groups. The decline in body weights was milder in mice of the LZ+CPT group than the CPT group. One-way ANOVA was used for statistical analysis. * indicates *P* < 0.05, compared with the control group; # indicates *P* < 0.05, compared with the CPT group. n = 5 mice/group.

Weight loss is an important indicator for intestinal injury in mice, and body weight changes after intraperitoneal administration of CPT-11 or PBS was illustrated in Figure 4. There were no apparent changes of body weight profiles in the control and LZ groups. In contrast, body weights decreased significantly with repeated CPT-11 injections in mice of the CPT and LZ+CPT groups. Compare to the control group, weight loss became significant after only two injections of CPT-11 in both groups (*P* < 0.05). Of note, the decline in body weights was milder in mice treated with LZ-8 compared to those receiving oral PBS in the CPT group, reaching a significant difference on day 9 (0.87 ± 0.04 vs 0.79 ± 0.02; *P* = 0.031). Our data signified the alleviating effect of LZ-8 on body weight loss in mice with CPT-11-induced intestinal injury.

### Alleviation of intestinal injury in LZ-8-treated mice after repeated CPT-11 injections

**Figure 5:**
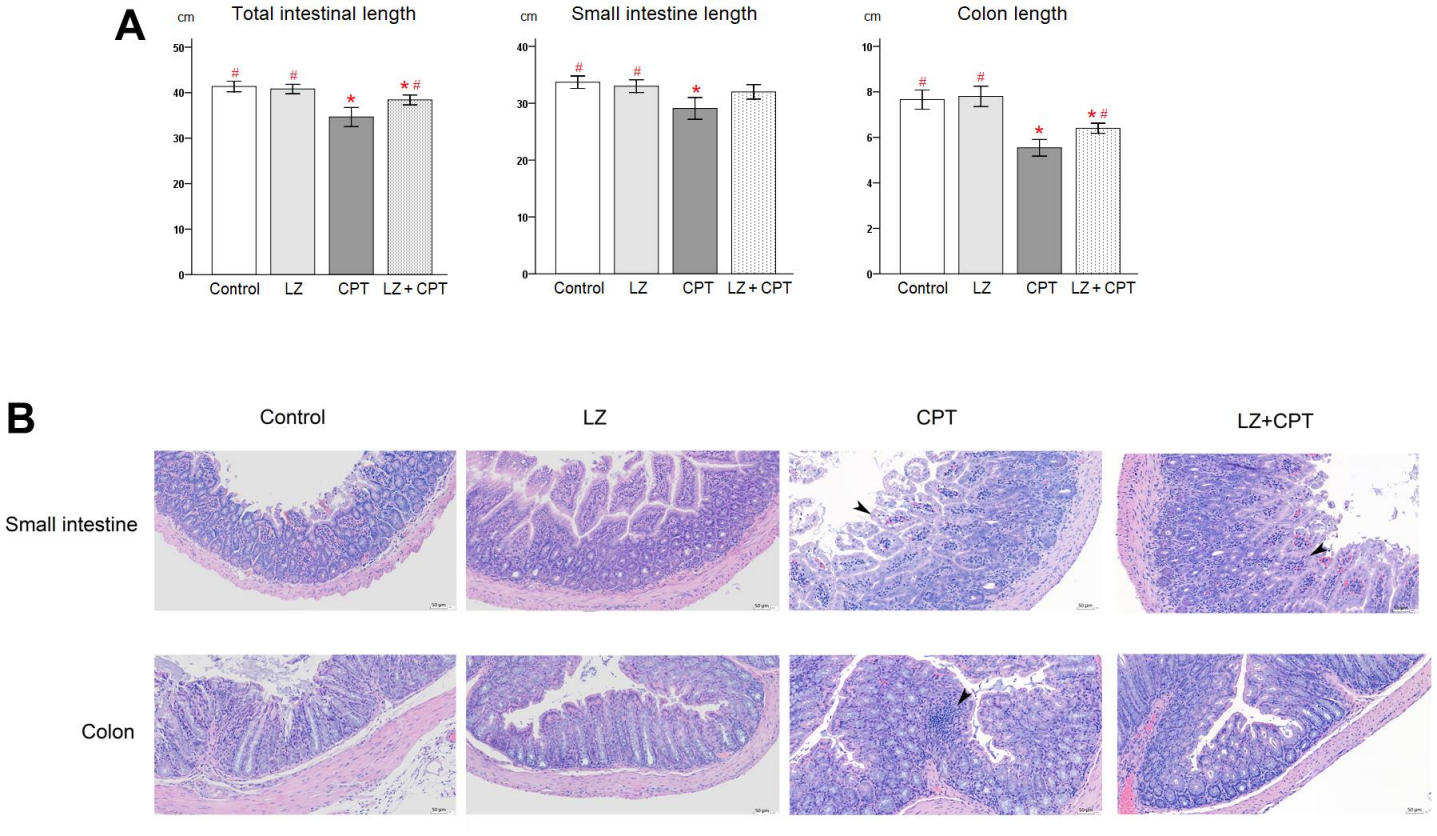
**Protective effects of LZ-8 on intestinal damage by CPT-11 in mice.** (**A**) Lengths of the total intestine, small intestine, and colon in mice following different treatments. No obvious changes of the intestinal lengths were observed in mice of the LZ group, but significant shortening of the intestinal lengths was noted in mice of the CPT group. The reduction in intestinal lengths was ameliorated in mice of the LZ+CPT group, compared to the CPT group. Data were presented as mean ± SD, and one-way ANOVA was used for statistical analysis. * indicates *P* < 0.05, compared with the control group; # indicates *P* < 0.05, compared with the CPT group. n = 5 mice/group. (**B**) Microscopic histopathology of the small intestine and colon tissue sections from mice with different treatments (H&E staining, 200×). Normal intestinal architecture was noted in the control and LZ groups. The small intestine section of the CPT and LZ+CPT groups showed an edematous change of the intestinal villi with hyperemia (arrowhead) and prominent infiltration of inflammatory cells into the lamina propria. The major abnormal finding in the colon section was the increase of inflammatory cells in the lamina propria (arrowhead). Compared to the CPT group, the histopathologic alterations were attenuate in the LZ+CPT group, with nearly normal architecture of the colon.

As a strong correlation of length reduction with murine intestinal injury, intestinal lengths were measured immediately after sacrifice. Compared to the control group, no obvious changes of the intestinal lengths were observed in mice of the LZ group (Figure 5A). On the other hand, total lengths of the intestine shortened significantly in mice of the CPT group (*P* = 0.003). The significant shortening of lengths in the CPT group can be observed in both small intestine and colon (*P* = 0.012 and *P* < 0.001, respectively). It is worth to note that the reduction in intestinal lengths was ameliorated in mice of the LZ+CPT group, compared to the CPT group. The influence of LZ treatment was more evident on the lengths of the total intestine and colon (*P* = 0.045 and *P* = 0.013, respectively).

Hematoxylin and eosin staining revealed normal architecture of the small intestine and colon in the control and LZ groups (Figure 5B). On the contrary, several histopathologic changes indicating intestinal injury were found in the CPT group, including rupture of epithelial cells, goblet cell depletion, irregularly arranged glands, edema, and infiltration of inflammatory cells into the lamina propria. The alterations were more significant in the small intestine than in the colon. Notably, the histopathologic alterations in both small intestine and colon were attenuate in mice of the LZ+CPT group, compared to the CPT group.

### Restoration of claudin-1 expression in the intestinal membranes by LZ-8 treatment in mice with CPT-11-induced intestinal injury

**Figure 6:**
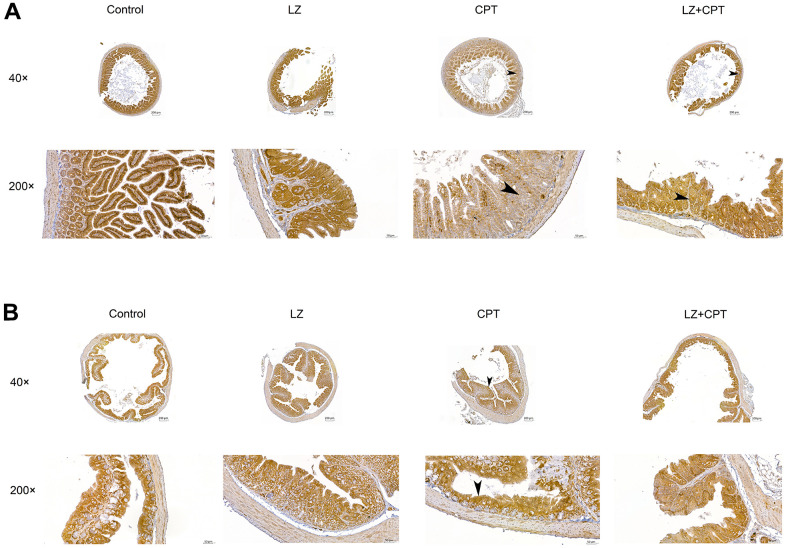
**Restoration of claudin-1 expression in the intestinal membranes by LZ-8 treatment in mice with CPT-11-induced intestinal injury, assessed by immunohistochemical staining.** (**A**) The small intestine sections (40× and 200×). (**B**) The colon sections (40× and 200×). Homogeneous strong membrane staining (3+) was noted in the control and LZ groups. The intensity diminished in the CPT group, only 1+ in the small intestine section (arrowhead) and 2+ in the colon section (arrowhead). Compared to the CPT group, the intensity increased in the LZ+CPT group, showing 2+ in the small intestine section (arrowhead) and 3+ in the colon section (arrowhead).

In the *in vitro* experiments, we found that pretreatment of LZ-8 could improve the expression of claudin-1 in IEC-6 cells following CPT-11 treatment and provide protective effects on CPT-11-induced cytotoxicity. Further, we examined if claudin-1 also involved in the effects of LZ-8 on mice with CPT-11-induced intestinal injury. As shown in Figure 6, the expression of claudin-1 in the intestine in mice receiving different treatments was detected by immunohistochemical assay. Strong membrane staining (3+) was noted in the small intestine and colon tissue sections from mice of the control and LZ groups. The intensity diminished, only 1+ in the small intestine sections and 2+ in the colon sections, in the CPT group. It is of value to observe that the intensity of membrane staining for claudin-1 increased to 2+ and 3+ in the small intestine and colon sections from mice of the LZ+CPT group, respectively. Along with the histopathologic findings, these results suggested that LZ-8 treatment could restore the expression of claudin-1 in the intestinal membranes in mice with CPT-11-induced intestinal injury.

## DISCUSSION

CPT-11 is a valuable chemotherapeutic agent in treating a variety of solid tumors. Potential intestinal toxicity is the main limiting factor in its clinical use. In the present study, cell death in a dose-dependent manner was demonstrated in IEC-6 cells treated with CPT-11. Meanwhile, we found that the impact of CPT-11 on the viability of IEC-6 cells was augmented markedly as an increase of the incubation time. Accordingly, patients with predisposing factors associated with an increase in the half-life of CPT-11, such as liver dysfunction, should be treated with caution.

The pathophysiology of CPT-11-induced intestinal injury is complex, and implicated factors include direct damages to the epithelium, inflammatory cell infiltration, and bacterial dysbiosis [9]. In the present study, we demonstrated histopathologic changes in the intestine from mice with CPT-11-induced intestinal injury. Consistent with previous reports, the alterations were observed in both small intestine and colon [10, 11], but we found more evident in the small intestine. Some molecular mechanisms underlying CPT-11-induced intestinal injury were proposed. Several studies indicated that CPT-11 triggered high production levels of inflammatory cytokines, such as tumor necrosis factor-α, interleukin-6, and interleukin-1β, in the intestine tissues [12–14]. Increased levels of cyclooxygenase-2 and prostaglandin E-2 were observed in rat colon following CPT-11 administration [15]. Some investigators indicated that inflammation by CPT-11 was through activation of NF-κB signaling [14, 16]. Upregulated expression of endoplasmic reticulum stress-associated proteins, such as P4HB and PRDX4, was also implicated to be associated with CPT-11-induced intestinal injury [16]. Aside from the aspect of inflammatory induction, the present study demonstrated the involvement of tight junction in the intestinal damage by CPT-11. Downregulated expression of claudin-1 in a dose-dependent manner was observed in CPT-11-treated IEC-6 cells. In mice with CPT-11-induced intestinal injury, the expression of claudin-1 in both small intestine and colon was significant decreased. Together with results of the viability study and histopathologic assessment, claudin-1 may play a role in the intestinal damage by CPT-11.

Tight junctions, consisting of a network of anastomosing strands, are epithelial intercellular junctions located at the most apical region of cell-cell contacts [17]. These structures are responsible for sealing the intercellular space and regulating the paracellular permeability. The family of claudins constitute the major components of tight junction strands and form size- and charge-specific pores to regulate tissue-specific permeability [18]. Among them, claudin-1 plays the fundamental role in sealing the paracellular space for the maintenance of epithelial barrier integrity [19]. Therefore, alterations in its expression may lead to the disturbance in gut homeostasis and are common in many pathological conditions. In murine models of intestinal injury, CPT-11 was found to cause intestinal tight junction damage which can lead to bacterial translocation and the occurrence of diarrhea [20, 21]. In the present study, downregulated expression of claudin-1 by CPT-11 was demonstrated in both IEC-6 cells and intestinal membranes of mice. As a result, the weakening of intestinal barriers may lead to increased paracellular transport of solutes and water that causes leak-flux diarrhea, as observed in the present animal study.

Severe diarrhea is the major symptom of gastrointestinal toxicity that limits the clinical use of CPT-11. Several approaches have been proposed to ameliorate the side effect, but none shows sufficient efficacy. The use of herbal medicine is increasing worldwide, and a large variety of plant extracts essentially derived from traditional medicine have been characterized for their capacity to combat CPT-11-induced diarrhea [3, 22]. In the present study, we found that LZ-8-treated IEC-6 cells were more resistant to CPT-11-induced damage. In our animal study, LZ-8 could mitigate CPT-11-associated symptoms and intestinal damage in mice. Accordingly, LZ-8 may be a potential candidate for reducing CPT-11-induced intestinal toxicity.

Despite numerous studies regarding a variety of biological activities of LZ-8 have been reported [4, 5], limited data are available on its protection against intestinal injury. Oral administration of *Ganoderma lucidum* polysaccharides was documented to improve intestinal barrier function in rats [23]. LZ-8 is a fungal immunomodulatory protein in *Ganoderma lucidum* mycelia [6], but it is still not known whether LZ-8 exerts its protective function via regulating intestinal epithelial barrier. In our previous work, LZ-8 was demonstrated to protect IEC cells against inflammation-induced injury *in vitro* and to attenuate symptoms and inflammatory responses of dextran sulfate sodium-induced colitis in mice [24]. The mechanism of its protective effects on IEC cells was related to barrier function improvement by enhancing transepithelial electrical resistance, reducing permeability, and maintaining distribution of tight junction proteins. In the present study, the expression levels of claudin-1 were elevated if CPT-11-treated IEC-6 cells were pretreated with LZ-8. Meanwhile, the intensity of membrane staining for claudin-1 in the intestine was significantly increased in LZ-8-treated mice following CPT-11-induced intestinal injury. Along with results of the viability study and histopathologic assessment, these results implicated that LZ-8 may exert the protective effects against CPT-11 damage through restoring claudin-1 expression in intestinal cells. Taken together, tight junction regulation may be a new function of LZ-8. As CPT-11 is an important chemotherapeutic agent to treat various solid tumors, LZ-8 might be useful in the management of intestinal toxicity induced by chemotherapy.

Collectively, LZ-8 displayed beneficial effects against CPT-11 damage in both IEC-6 cells and mice. CPT-11 treatment was accompanied by downregulated expression of claudin-1 and LZ-8 can restore claudin-1 expression in intestinal cells following CPT-11 treatment, suggesting the role of claudin-1 in the scenario. Additionally, it is important to note that no evident cytotoxicity of LZ-8 at all doses was observed in IEC-6 cells. In summary, our data provided a potential strategy to apply LZ-8 in the treatment of CPT-11-associated gastrointestinal adverse effects in humans.

## MATERIALS AND METHODS

### Chemicals and reagents

CPT-11 (Irinotecan) was purchased from the Pfizer Pty Limited (Perth, Australia). LZ-8 was isolated from LZDCH (Yeastern Biotech, Taipei, Taiwan), which was extracted form *Ganoderma lucidum*, as described previously [24, 25]. Briefly, LZDCH was dissolved and centrifuged, and proteins were obtained by filtration. LZ-8 was purified by fast protein liquid chromatography (GE Healthcare, Chicago, IL, USA).

### *In vitro* CPT-11-induced damage to IEC-6 cells

### Cell preparation

IEC-6, the normal rat small intestine epithelial cell line, was obtained from the American Type Culture Collection (ATCC CRL-1592, Manassas, VA, USA). IEC-6 cells were cultured in Dulbecco’s modified Eagle’s medium (Gibco/Thermo Fisher Scientific, Waltham, MA, USA) supplemented with 10% fetal bovine serum, 0.1 unit/ml insulin, and 1% penicillin-streptomycin. These cells were incubated at 37° C in a humidified atmosphere under 5% CO_2_.

### Evaluation of cell viability

IEC-6 cells were seeded in 96-well plates at a density of 4 × 10^3^ cell/well and incubated for 24 hours. To investigate cell viability of IEC-6 cells after LZ-8 or CPT-11 treatment, cells were exposed to various concentrations of LZ-8 (1, 3, and 10 μg/ml) or CPT-11 (10, 20, and 50 μg/ml) for 24 or 48 hours. Then, CCK-8 reagent (Sigma-Aldrich, St. Louis, MO, USA) was added to each well followed by incubation for a period of 2 hours at 37° C. The absorbance was measured by spectrophotometry (BioTek Instruments, Santa Clara, CA, USA) at 450 nm. For control, the absorbance of untreated cells was considered 100%.

To evaluate protective effects of LZ-8 on CPT-11-induced damage to IEC-6, IEC-6 cells were pretreated with 10 μg/ml LZ-8 for 24 hours. The medium was then removed and replaced with fresh medium containing 50 μg/ml CPT-11. The cells were continuously treated for 24 or 48 hours. Subsequently, CCK-8 colorimetric assay was used for quantitation of the viable cell number, as stated above.

### Determination of claudin-1 expression by qPCR and Western blotting

As tight junctions are essential to maintain intestinal barrier integrity, qPCR and Western blotting were used to determine the expression of claudin-1 in IEC-6 cells following different treatments. As above, IEC-6 cells were exposed to various concentrations of LZ-8 or CPT-11 for 24 hours. To assess the influence of LZ-8 pretreatment on CPT-11-induced damage, IEC-6 cells were treated with 10 μg/ml LZ-8 for 24 hours and subsequent 50 μg/ml CPT-11 for 24 hours. After indicated treatments, the cells were harvested for further experiments.

For qPCR, total RNA was extracted using Total RNA Mini Kit (Geneaid, New Taipei City, Taiwan). Following extraction, concentrations of RNA samples were measured spectrophotometrically at the optical density of 260/280 (NanoDrop Technologies, Wilmington, DE, USA). cDNA was then synthesized using cDNA Reverse Transcription Kit (Applied Biosystems, Foster City, CA, USA). The expression of β-actin was used as the internal control. The sequences of primers were as follows: claudin-1, forward 5’-CTGGGAGGTGCCCTACTTT-3’ and reverse 5’-CCGCTGTCACACGTAGTCTT-3’; β-actin, forward 5’-GTCAGGTCATCACTATCGGC-3’ and reverse 5’-CATGGATGCCACAGGATTCC-3’. According to the manufacturer’s instructions, qPCR was performed using cDNA samples with SYBR Green PCR Master Mix on the ABI 7300 Real-time PCR system (Applied Biosystems, Foster City, CA, USA).

For Western blot analysis, harvested IEC-6 cells were lysed with PRO-PREP™ Protein Extraction Kit (iNtRON Biotechnology, Seongnam, Korea). The equal amounts of proteins were fractionated on sodium dodecyl sulfate-polyacrylamide gel electrophoresis and transferred to polyvinylidene fluoride membranes. After blocking with 5% skimmed milk in Tris-buffered saline with 0.1% Tween 20, the membranes were incubated with primary antibodies, anti-claudin-1 (2H10D10, #37-4900, Gibco/Thermo Fisher Scientific, Waltham, MA, USA) or anti-β-actin (AC-74, A2228, Sigma-Aldrich, St. Louis, MO, USA), at 4° C overnight. Then, the membranes reacted with horseradish peroxidase-conjugated secondary antibodies (Sigma-Aldrich, St. Louis, MO, USA) at room temperature for 1 hour. The signals were developed by an enhanced chemiluminescence kit (Sigma-Aldrich, St. Louis, MO, USA) and visualized by an imaging system. The intensity of bands was measured by the AlphaImager 2000 Analysis System (Alpha Innotech, San Leandro, CA, USA).

### CPT-11-induced intestinal injury in mice

### Animals and treatments

**Figure 7:**
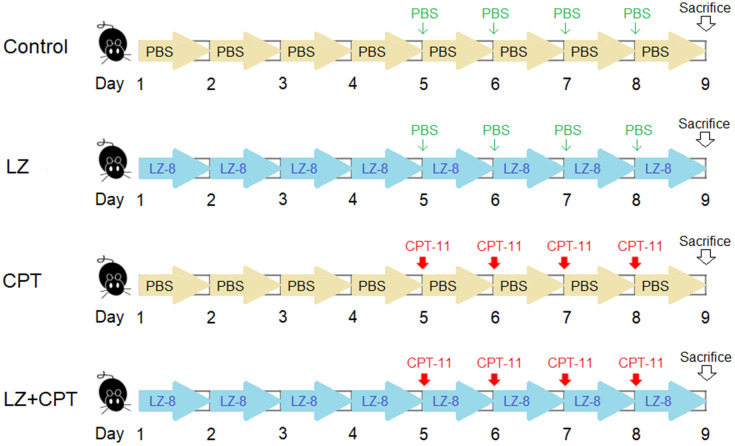
**The dosing schedule of CPT-11 and LZ-8 in the animal study of CPT-11-induced intestinal injury.** n = 5 mice/group.

Eight-week-old female Balb/c mice weighing 20 ± 2 g were obtained from the National Laboratory Animal Center (Taipei, Taiwan) and maintained in a temperature- and humidity-controlled environment with free access to food and water in the Laboratory Animal Center of the Chung Shan Medical University. Mice were randomly divided into four groups with five mice in each group (Figure 7). 50 μg LZ-8 in 100 μL of PBS was given to mice of the LZ and LZ+CPT groups through gavage once a day for 8 days. Mice of the control and CPT groups were daily fed with PBS (100 μL) for control. To induce intestinal injury, mice of the CPT and LZ+CPT groups received intraperitoneally injections of CPT-11 (75 mg/kg) for 4 consecutive days, starting from day 5 to day 8. Mice of the control and LZ groups were injected with PBS at the same time points for control.

### Assessment

All mice were monitored for feces conditions throughout the study period, and scoring for diarrhea was conducted daily. The severity of diarrhea was scored as follows: 0, normal; 1, soft feces or small black feces; 2, wet and unformed feces; 3, watery feces with perianal staining of the coat [15]. Fecal occult blood was determined by the S-Y Occult Blood Reagent (Shin-Yung Medical Instruments Co., Taipei, Taiwan) and scored from 0 to 4+ (0 = negative, 4+ = most severe), according to the manufacturer’s instructions. As weight loss is a sufficient and economical parameter of murine intestinal injury, body weights of all mice were measured daily throughout the experimental period.

### Intestinal histopathology and immunohistochemistry

On day 9, all mice were anesthetized by intraperitoneal injection of the mixture of anesthetic agents (25 mg/kg Zoletil 50 and 7.5 mg/kg xylazine). After deep anesthesia, the concentration of CO_2_ in the cage increased gradually and the mice were euthanized. Then, intestinal samples were isolated immediately. The lengths of the total intestine, small intestine, and colon of each mouse were determined. The intestinal samples were immersed in 10% neutral buffered formalin for 24 hours. After fixation, the specimens were embedded in paraffin, sectioned about 5 μm thick, and stained with hematoxylin and eosin for histopathologic assessment.

For immunohistochemical analysis, the intestinal tissue sections from deparaffinized specimens were stained with anti-claudin-1 antibody (2H10D10, Cat #37-4900, Gibco/Thermo Fisher Scientific, Waltham, MA, USA). The UltraVision Quanto Detection System HRP was used to amplify the signal. The immunostaining was visualized with DAB Quanto Chromogen and Substrate (Thermo Fisher Scientific, Waltham, MA, USA) and counterstained with hematoxylin. Stained tissue sections were reviewed by a pathologist, and the membrane staining intensity was estimated as stated below: strong (3+), recognizable thick membranes and dark brown membrane staining with 4× objective lens; moderate (2+), recognizable thick membranes and brown membrane staining with 4× or 10× objective lens; weak (1+), thin membranes and light brown membrane staining with 10× or 20× objective lens [26].

### Statistical analysis

Data analysis was performed using SPSS 16.0 for Windows. For continuous variables, statistical comparisons of the different treatment groups were carried out by one-way ANOVA, with Games-Howell test for post hoc analysis. For diarrhea score and occult blood score, chi-square test was used to compare the groups. A value of *P* < 0.05 was considered statistically significant.

## Supplementary Material

Supplementary Figure 1
