# Supplementary figures and images for: Ling Zhi-8, a fungal immunomodulatory protein in Ganoderma lucidum, alleviates CPT-11-induced intestinal injury via restoring claudin-1 expression
Source: Aging (Albany NY). 2023 May 5;15(9):3621–34. doi: 10.18632/aging.204695 (PMC10449289; doi:10.18632/aging.204695)

SUPPLEMENTARY FIGURE

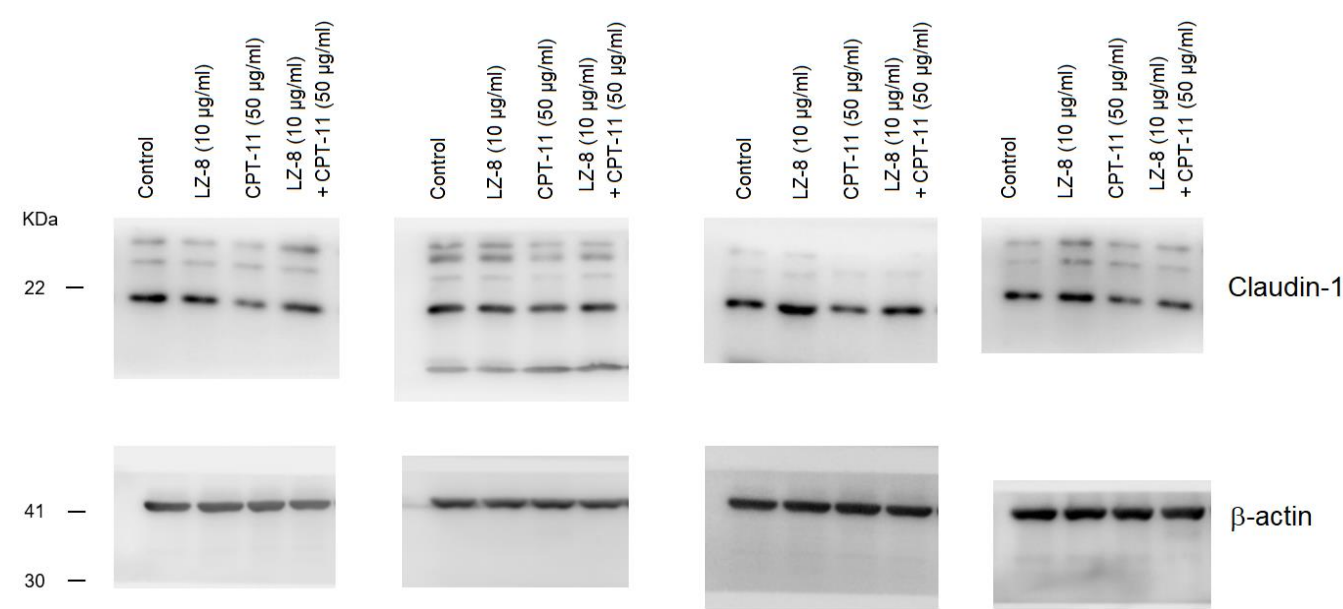

Supplementary Figure 1. Full, uncropped Western blots.

Supplement: Supplementary Figure 1 [file aging-15-204695-s001.pdf]
